# The Cunning Airway: Anesthetic Insights From a Five-Case Series on Intubation Challenges Without Tracheostomy in Catastrophic Maxillofacial Injury

**DOI:** 10.7759/cureus.99712

**Published:** 2025-12-20

**Authors:** Sarita Kumari, Somsubhra Pal, Yashpal Singh, Akansha Dhilip

**Affiliations:** 1 Anaesthesiology, Institute of Medical Sciences, Banaras Hindu University, Varanasi, IND

**Keywords:** advanced airway management, airway access, airway management, airway procedures, awake fiberoptic intubation, difficult airway management, maxillofacial trauma, nasal intubation, submental intubation

## Abstract

Panfacial trauma involves concurrent injuries to the upper (frontal), mid-face (maxilla, zygoma, nose), and lower (mandible) thirds of the face. This creates a significant threat to airway patency due to anatomical distortion, bleeding, edema, and the risk of aspiration. Airway management in panfacial trauma is complex and requires a strategic, individualized approach. Any error in this process may lead to considerable morbidity and mortality in both prehospital and hospital settings and can also complicate subsequent fracture reconstruction. Rapid and appropriate airway control is crucial to prevent hypoxia, facilitate surgical repair, and support postoperative recovery. This case series includes patients with panfacial fractures who required surgical management and advanced airway techniques. Approaches used include awake fiberoptic nasotracheal intubation, awake fiberoptic orotracheal intubation, awake video-laryngoscope-guided orotracheal intubation, and submental intubation. Submental intubation was performed in Cases 1-4, and nasal intubation was used in Case 5 to provide optimal access for both anesthetists and surgeons. Each case is reviewed for clinical presentation, airway technique, intraoperative findings, and postoperative outcomes. The approach used helped achieve better patient outcomes, supported early recovery, and avoided the need for surgical airway access such as tracheostomy or cricothyroidotomy. The planning and management process highlights the importance of timely decision-making, the availability of advanced airway equipment, and the goal of avoiding a surgical airway whenever possible.

## Introduction

Panfacial trauma involves high-energy impact to the face, causing injury to two or more regions of the craniofacial skeleton: the frontal bone, the mid-face, and the occlusal unit [1]. In all such injuries, the Advanced Trauma Life Support (ATLS) guidelines [2] must be followed to resuscitate the patient. A primary survey should be completed, followed by a detailed secondary survey, after which definitive interventions can be undertaken. For an anesthetist, securing the airway in this setting can be an uphill task. According to Hutchison et al. [3], six main problems related to maxillofacial trauma can affect airway management. First, a fractured maxilla may be displaced posteroinferiorly, blocking the inclined plane of the skull base and causing nasal airway obstruction. Second, in bilateral anterior mandibular or mandibular symphyseal fractures, the tongue can lose its support and fall backward in a supine patient, obstructing the oropharynx. Third, foreign bodies such as teeth, vomitus, or hematoma may obstruct the airway at any level. Fourth, active hemorrhage can further compromise airway patency. Fifth, soft tissue swelling and edema, especially in the oral cavity or upper airway, can cause significant obstruction. Finally, maxillofacial trauma may be associated with laryngotracheal injury, leading to blockage or displacement around the epiglottis, arytenoid cartilages, or vocal cords, which can result in serious airway emergencies [3].

In panfacial trauma, routine oral or nasal intubation may not be feasible, and tracheostomy is often used as the definitive method of securing the airway. Tracheostomy is indicated when fiberoptic intubation is difficult or when prolonged postoperative mechanical ventilation is anticipated. Although it provides a secure airway, it involves surgically opening the trachea and carries risks such as bleeding, infection, tracheal stenosis, voice changes, and difficulties with decannulation. Cricothyroidotomy is an alternative for emergency surgical airway access but is unsuitable for prolonged surgeries or long-term ventilation, and it carries similar complications because it also involves opening the anterior neck. Submental intubation, introduced by Altemir in 1986 [4], has gained popularity as it allows unobstructed surgical access without the need for tracheostomy. This technique is supported by Jundt et al. [5] and Mishra et al. [6], who reported it as a safe and effective alternative in appropriate cases. In a study by Goh et al. [7] involving 2,229 patients, maxillofacial trauma was the indication for submental intubation in 81% of cases, with a mean procedure time of 10 minutes and a 7% complication rate, the most common being superficial dermal infection. Awake oral fiberoptic-guided intubation, with or without aids such as the Ovassapian airway, followed by submental tube placement, can secure the airway in patients with restricted mouth opening while maintaining surgical access [8]. If mouth opening permits passage of a laryngoscope, awake video-laryngoscope-guided orotracheal intubation can also be performed, provided adequate suctioning is done to clear blood or debris. This case series presents various scenarios in which airway management was achieved using different techniques, emphasizing the decision-making process and outcomes in each case. Patients with panfacial fractures who presented to our trauma center over a one-year period from September 1, 2024, to August 31, 2025, and who had no major comorbidities were included.

Ethics statement

Informed written consent for publication of clinical details and images was obtained from all patients. All efforts were made to maintain patient privacy.

## Case presentation

Case 1

**Figure 1:**
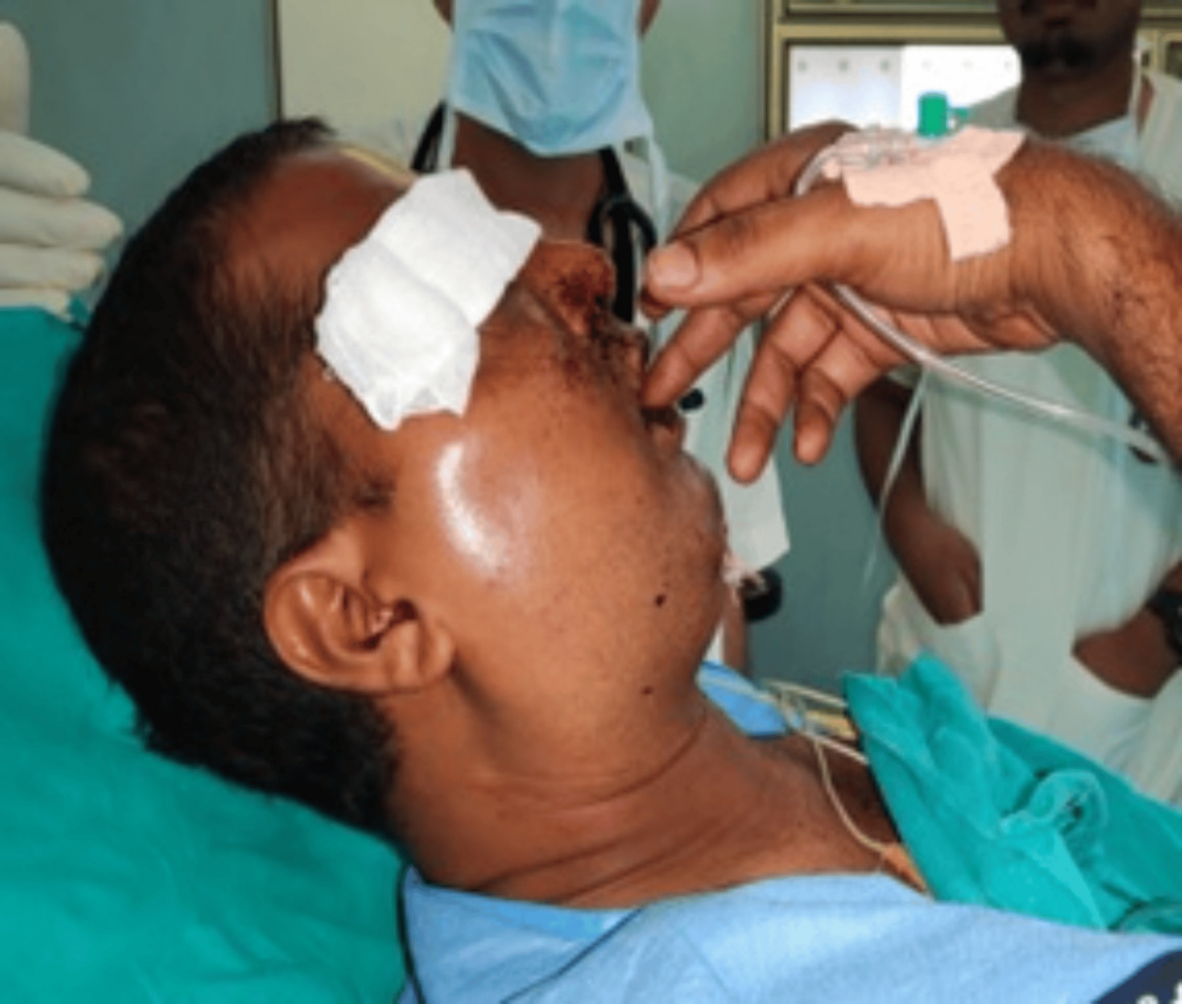
Restricted mouth opening (Case 1)

A 32-year-old male patient was brought to the trauma center following a roadside accident, with complaints of facial pain, swelling, and difficulty opening his eyes (Figure 1).

**Figure 2:**
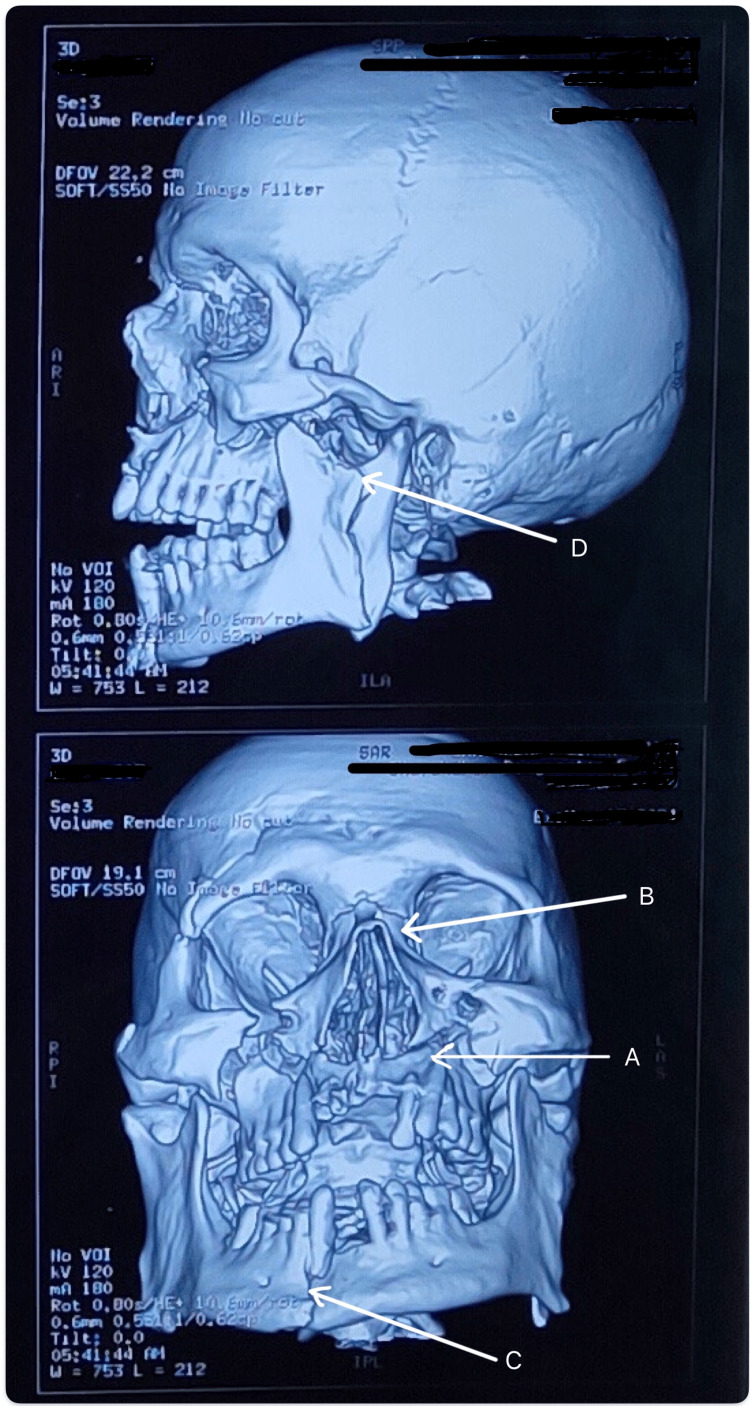
3D CT of the face and skull (Case 1) (A) LeFort type II fractures. (B) Fracture of the nasal process of the frontal bone. (C) Fracture of the right mandibular parasymphysis. (D) Fracture of the left mandibular subcondyle.

Non-contrast CT (NCCT) of the head with 3D reconstruction (Figure 2) revealed bilateral LeFort type II fractures [9] involving the nasal process of the frontal bone, the right mandibular parasymphysis, and the left mandibular subcondyle. On examination, the patient’s general and systemic assessments were unremarkable, with normal cardiovascular and respiratory findings, stable vital signs, a Glasgow Coma Scale (GCS) [10] score of E4V5M6, no focal neurological deficits, and a soft, non-tender abdomen. Airway assessment showed a patent airway but several significant challenges: facial edema, sutured lacerations over the chin and right eye, multiple loose and missing incisors, and severely restricted mouth opening (one finger breadth). Neck movements were normal. The patient was scheduled for maxillomandibular fixation (MMF) with open reduction and internal fixation (ORIF) under general anesthesia (GA). Significant airway difficulty was anticipated due to the risk of loose incisor dislodgement and potential bleeding. The primary airway strategy (Plan A) was awake oral fiberoptic intubation (AOFOI) [11], with emergency front-of-neck access (FONA) [12] designated as Plan B. The patient was counseled prior to the procedure and nebulized with 3 mL of 2% lignocaine with 1:200,000 adrenaline (LOX-ADR). Preoxygenation was done, and supplemental oxygen via nasal cannula was maintained throughout. Premedication consisted of IM glycopyrrolate 0.2 mg as an antisialagogue, IV midazolam 1 mg, and fentanyl 100 µg. Mild sedation was provided with a dexmedetomidine (DEX) infusion at 0.5 µg/kg/hr. Airway anesthesia was achieved using bilateral superior laryngeal nerve blocks (3 mL of 4% plain lignocaine per side), a trans-tracheal block (4 mL of 4% plain lignocaine), and 10% lignocaine spray to the oropharynx.

**Figure 3:**
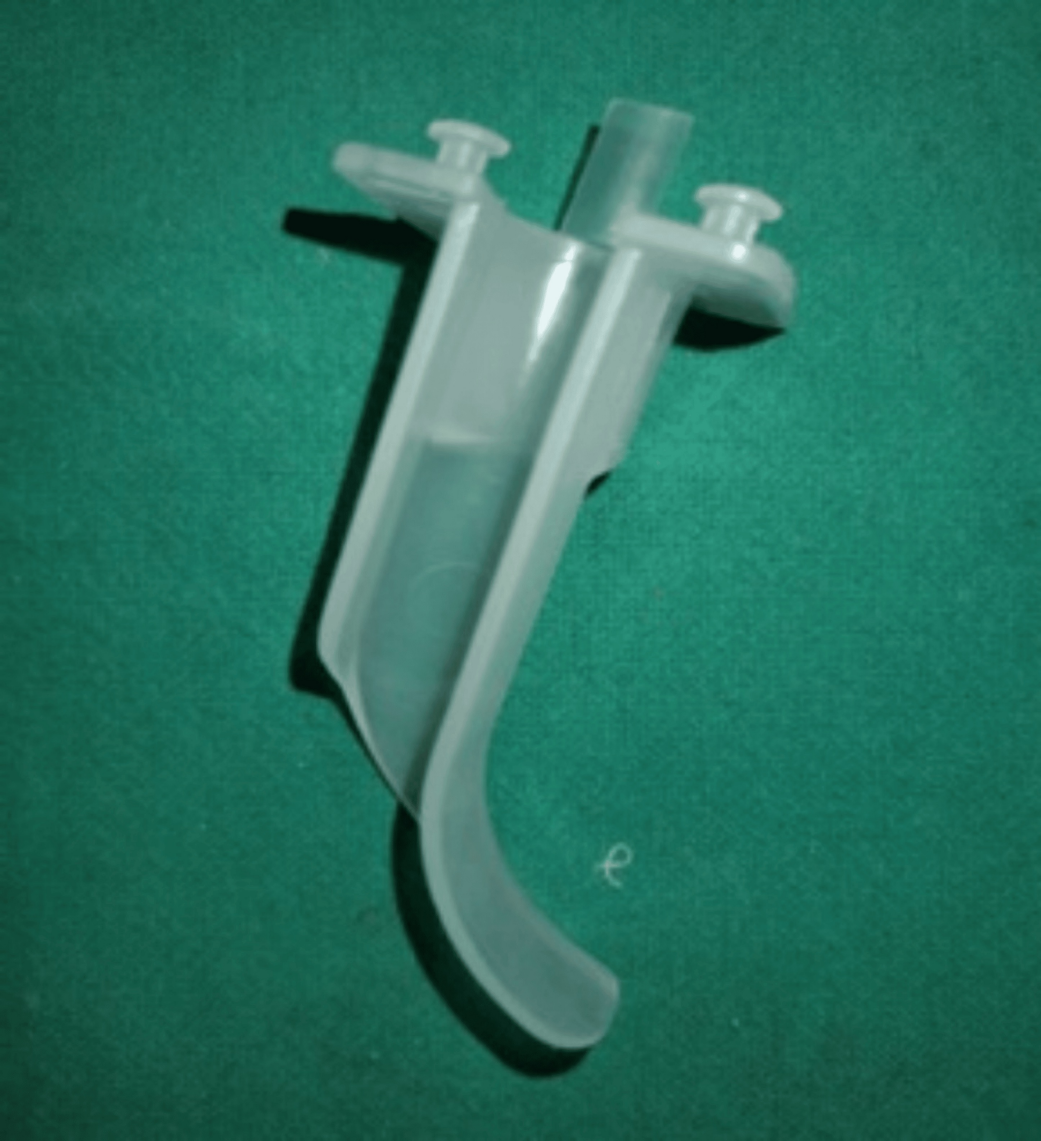
Ovassapian airway

**Figure 4:**
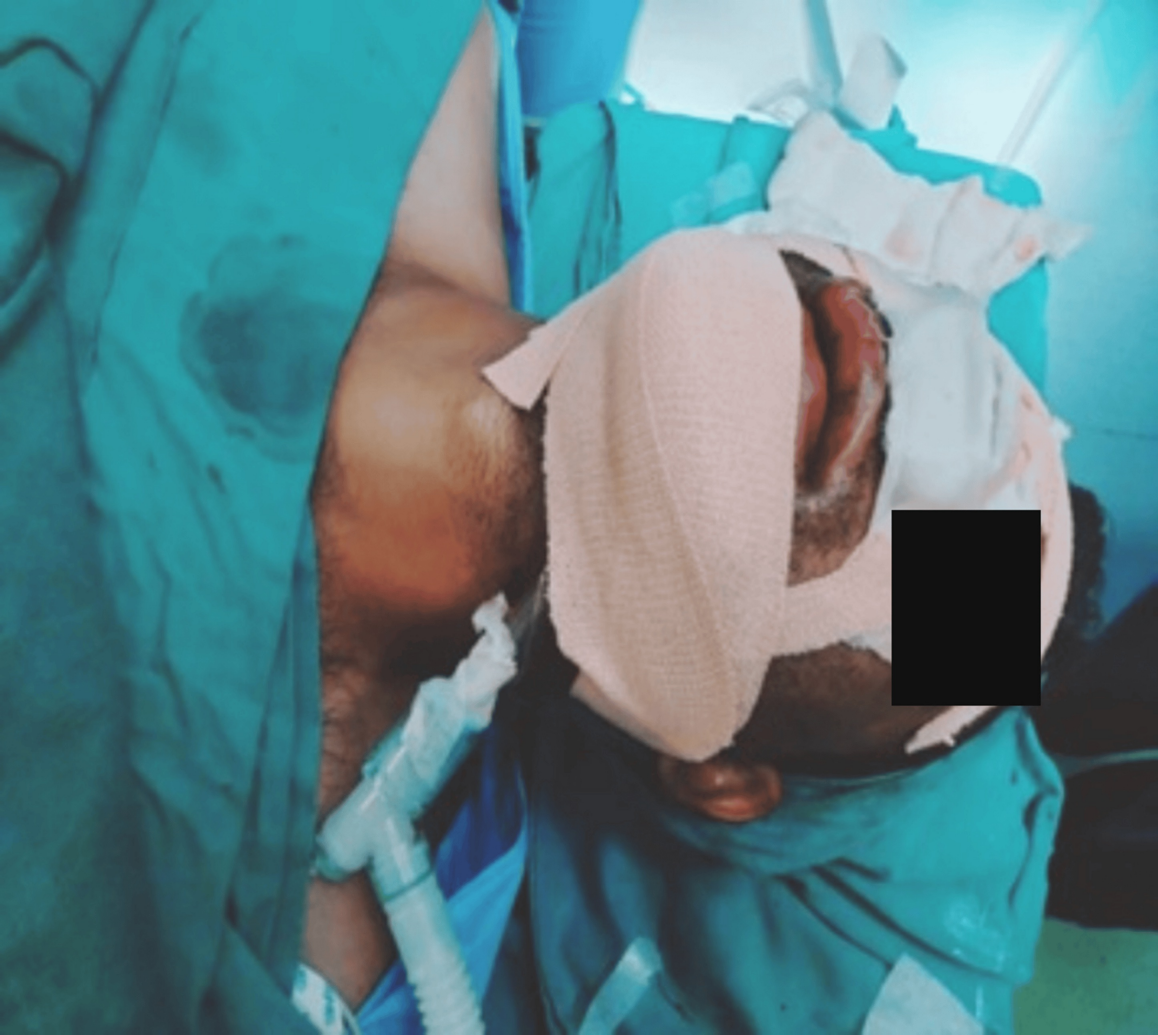
Endotracheal tube in submental position (Case 1)

**Figure 5:**
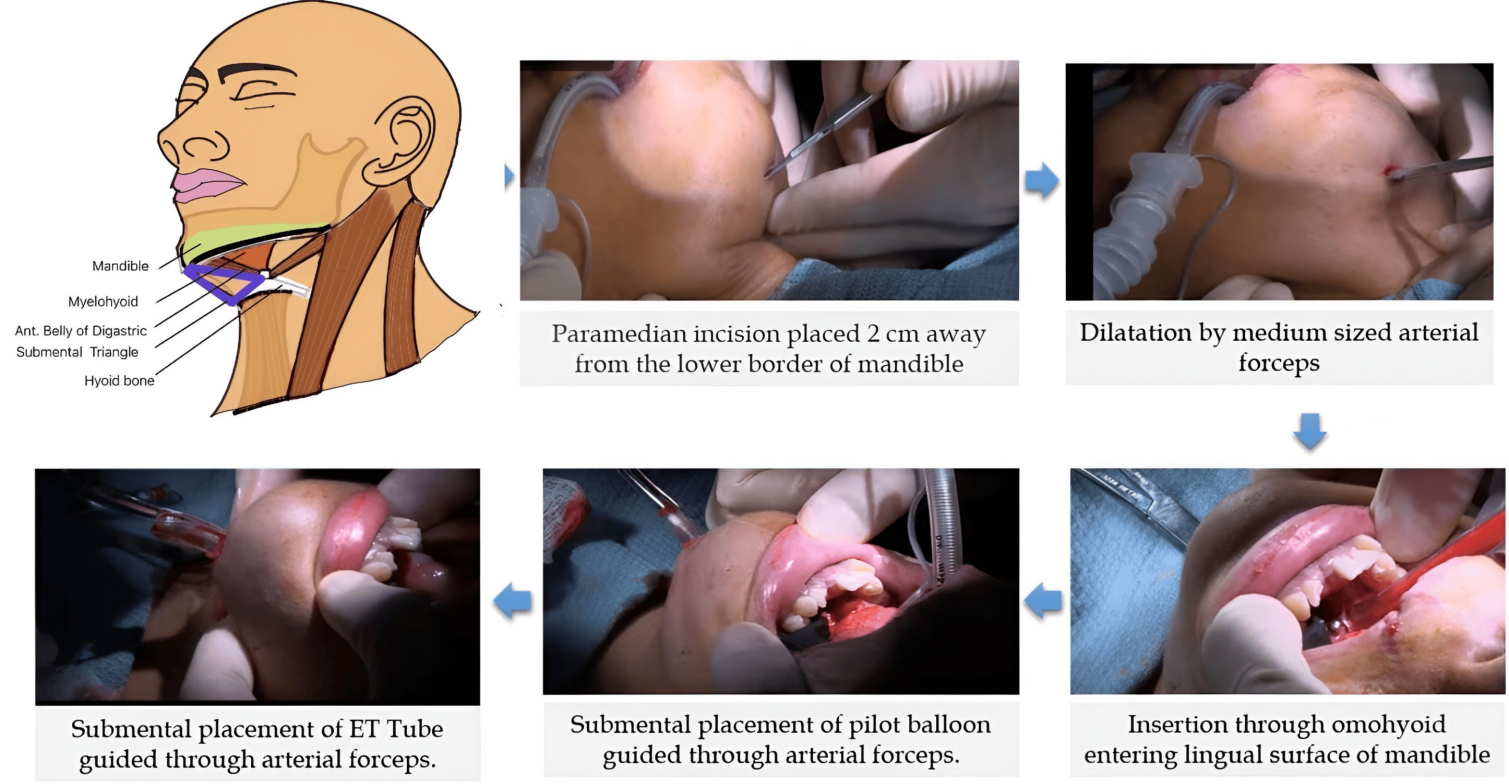
Submental intubation process The first image in this series is a schematic diagram showing the anatomy of the submental region (image credit: Dr. Sudipta Bera). The remaining images illustrate the step-by-step process of submental intubation.

Using an Ovassapian airway (Figure 3) placed in the oral cavity, a 7.5 mm I.D. flexometallic tube was inserted into the trachea, with its position confirmed visually and by capnography, followed by induction of anesthesia. Because submental placement was required, the skin over the submental area was scrubbed and draped in a sterile manner. A 2 cm incision was made near the lower border of the mandible, and blunt dissection was performed with artery forceps to create a tunnel toward the floor of the mouth. The overlying mucosa was then gently opened by blunt pressure. The endotracheal (ET) tube, with its connector removed, was grasped with the tip of the artery forceps and brought out through the submental region via the mucosal tunnel (Figures 4, 5).

After reattaching the ET tube connector, the ventilator circuit was connected to the tube, and its position was reconfirmed by auscultation and capnography. The tube was then secured in the submental position using a suture. Intubation was successful, and the intraoperative period remained uneventful. After completion of the planned procedure, the submental tube was returned to the oral position, and it was replaced with a PVC cuffed tube. The patient was reversed from anesthesia and transferred to the post-anesthesia care unit (PACU) with the tube in situ for airway preservation and overnight mechanical ventilation, given the long duration of surgery.

Case 2

**Figure 6:**
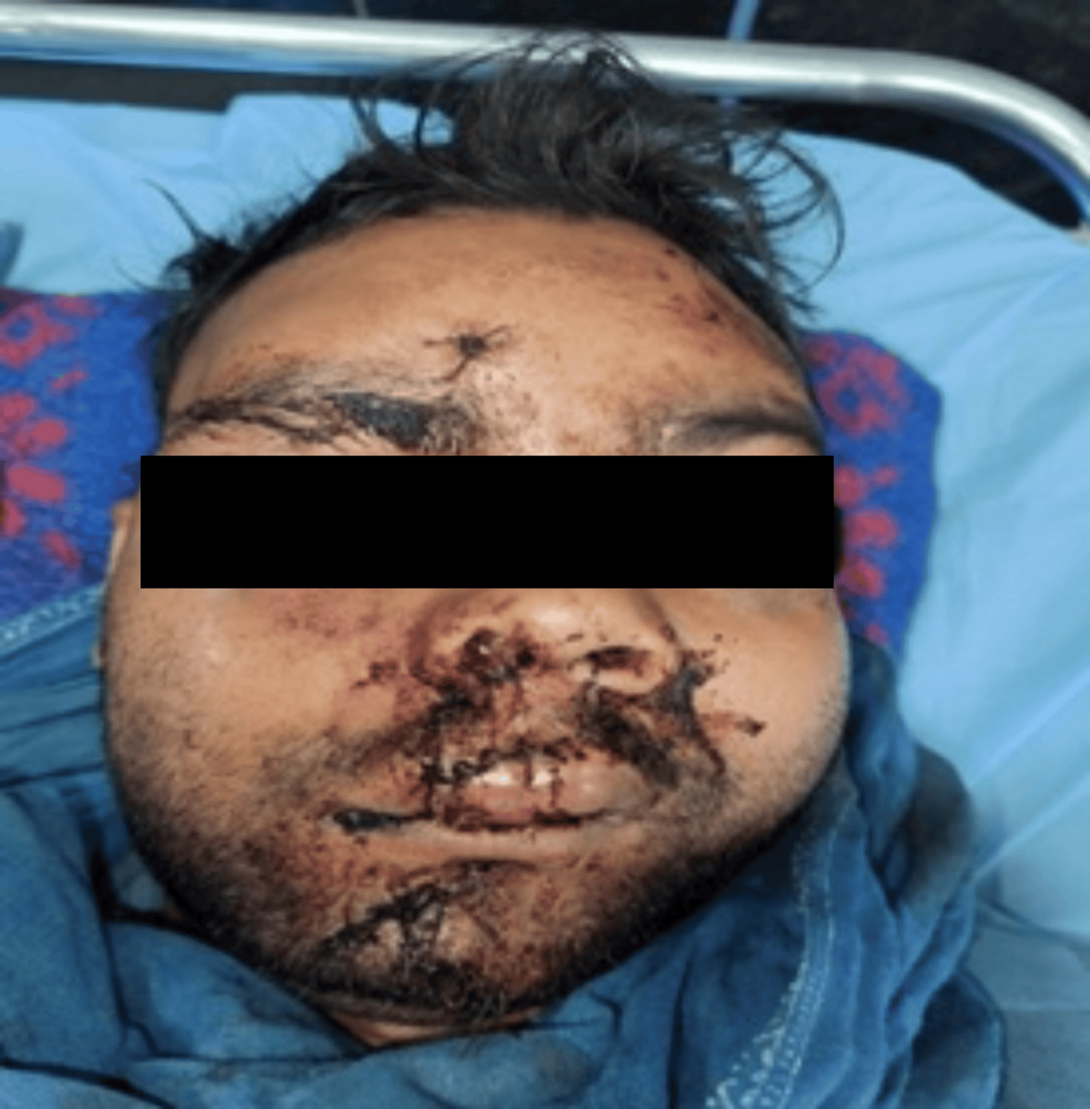
External facial injuries (Case 2)

A 29-year-old male patient presented to the trauma center triage following a road traffic accident, with complaints of facial pain and swelling (Figure 6).

**Figure 7:**
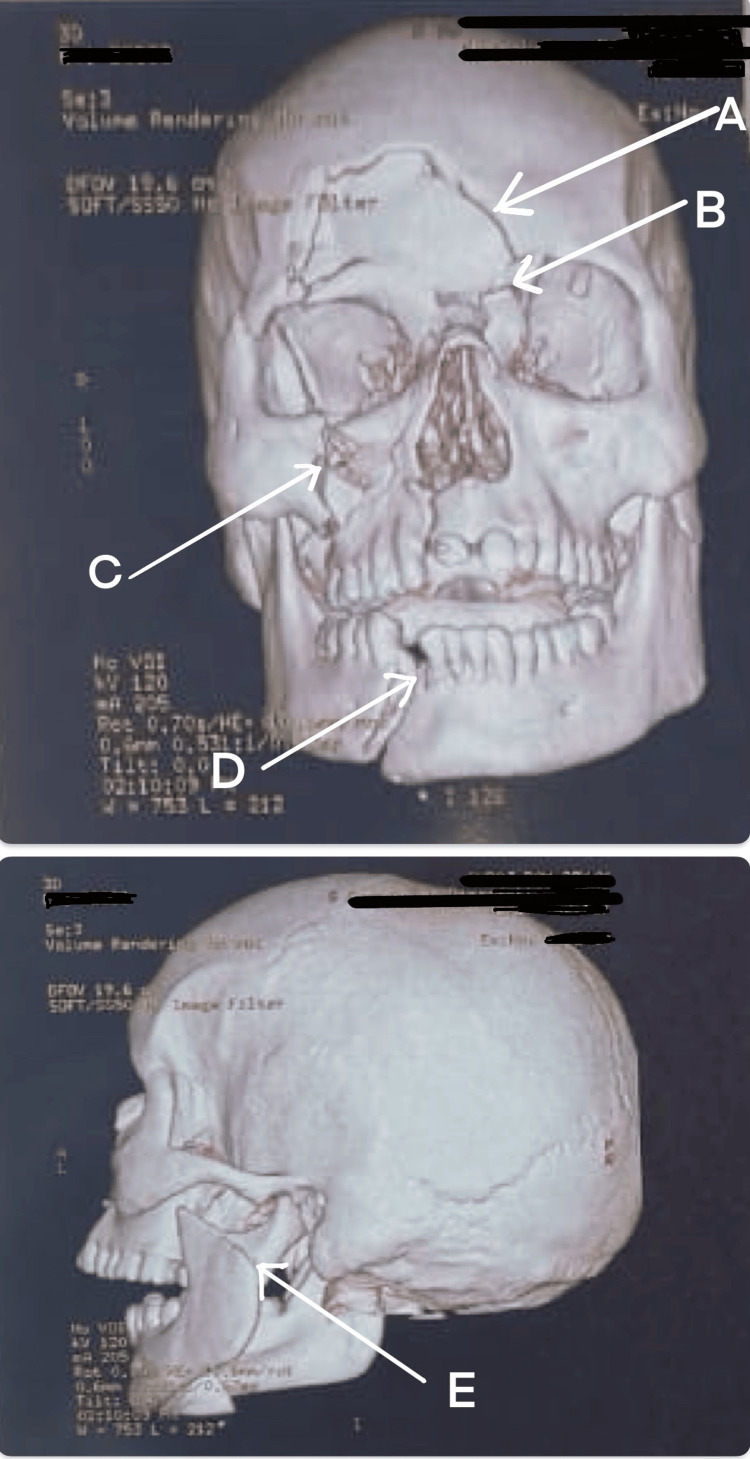
3D CT of the face and skull (Case 2) (A) Fracture of the right frontal bone. (B) Fracture of the left fronto-orbital suture. (C)Fracture of the right zygomaticomaxillary complex (ZMC). (D) Fracture of the right mandibular parasymphysis. (E) Fracture of the left condyle.

**Figure 8:**
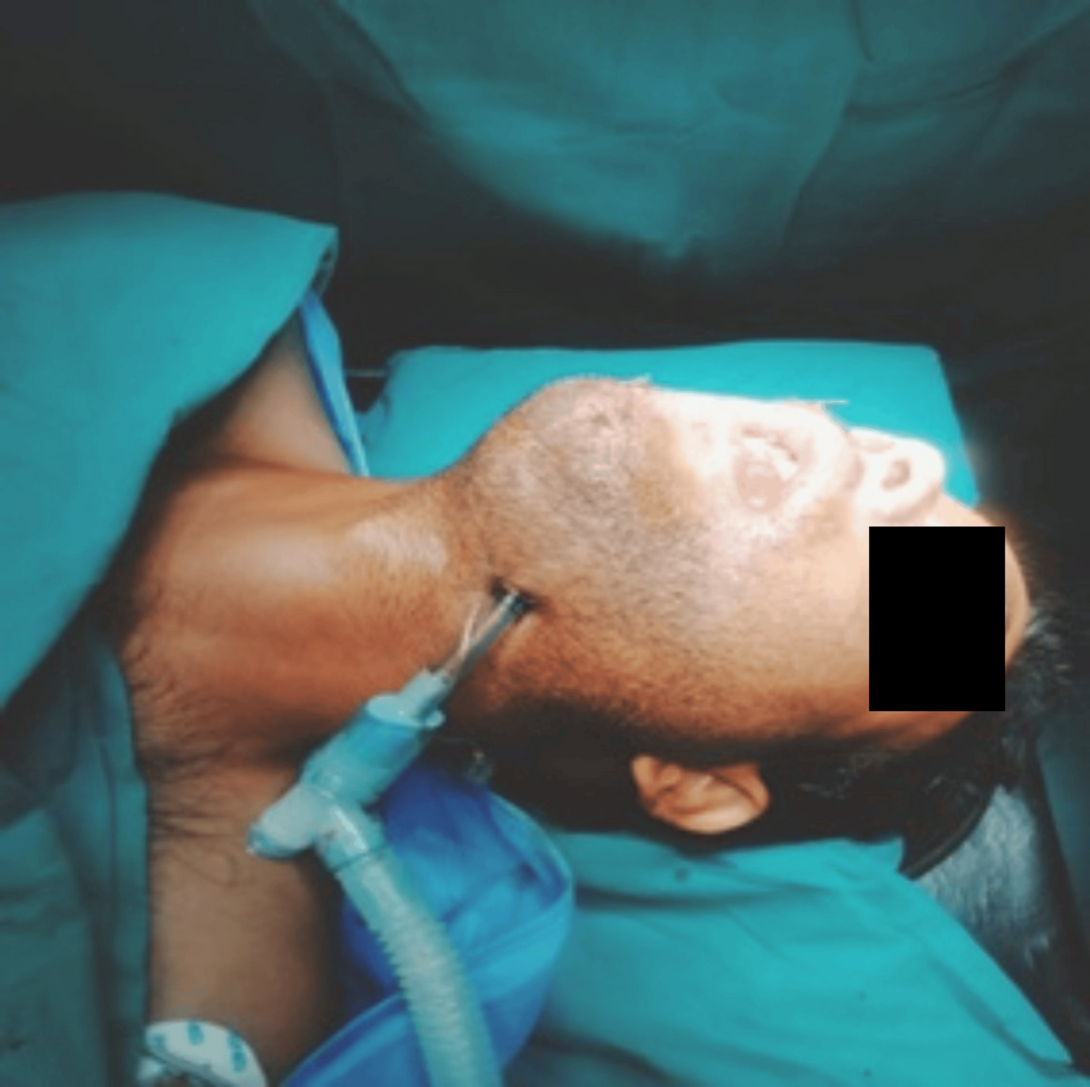
Endotracheal tube in submental position (Case 2)

3D CT imaging revealed fractures of the right frontal bone, left fronto-orbital suture, right zygomaticomaxillary complex (ZMC), right mandibular parasymphysis, and left condyle (Figure 7). The patient’s general and systemic assessments were unremarkable, with normal cardiovascular and respiratory findings and a GCS score of E4V5M6. Airway examination showed a patent airway despite facial and periorbital edema, lacerations over the upper lip and chin, multiple missing incisors, restricted mouth opening (two finger breadths), and a Mallampati Grade IV view [13,14]; neck movements were normal. The patient was scheduled for MMF with ORIF under GA, with an anticipated difficult airway primarily due to the risk of bleeding during intubation. The airway management plan included AOFOI as Plan A and emergency FONA as Plan B. Induction followed almost the same method used in Case 1, except dexmedetomidine infusion was avoided because of persistent bradycardia (HR ~50 to 55). AOFOI was successfully performed and converted to submental intubation using the previously described technique (Figure 8).

**Figure 9:**
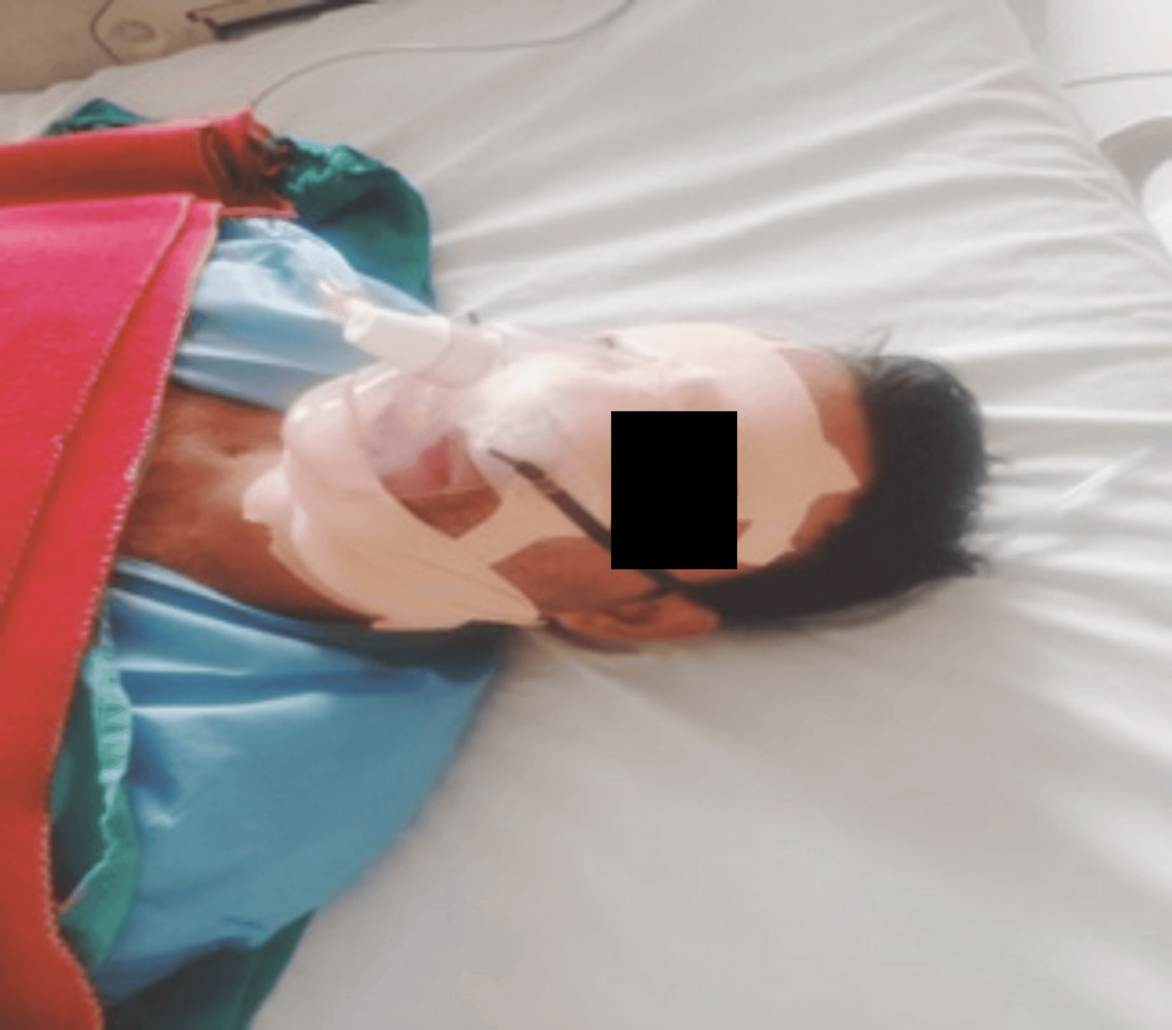
Patient post-extubation (Case 2)

The intubation and intraoperative period were uneventful. The patient recovered with minimal airway morbidity and was extubated the next day following precautionary overnight airway protection (Figure 9).

Case 3

**Figure 10:**
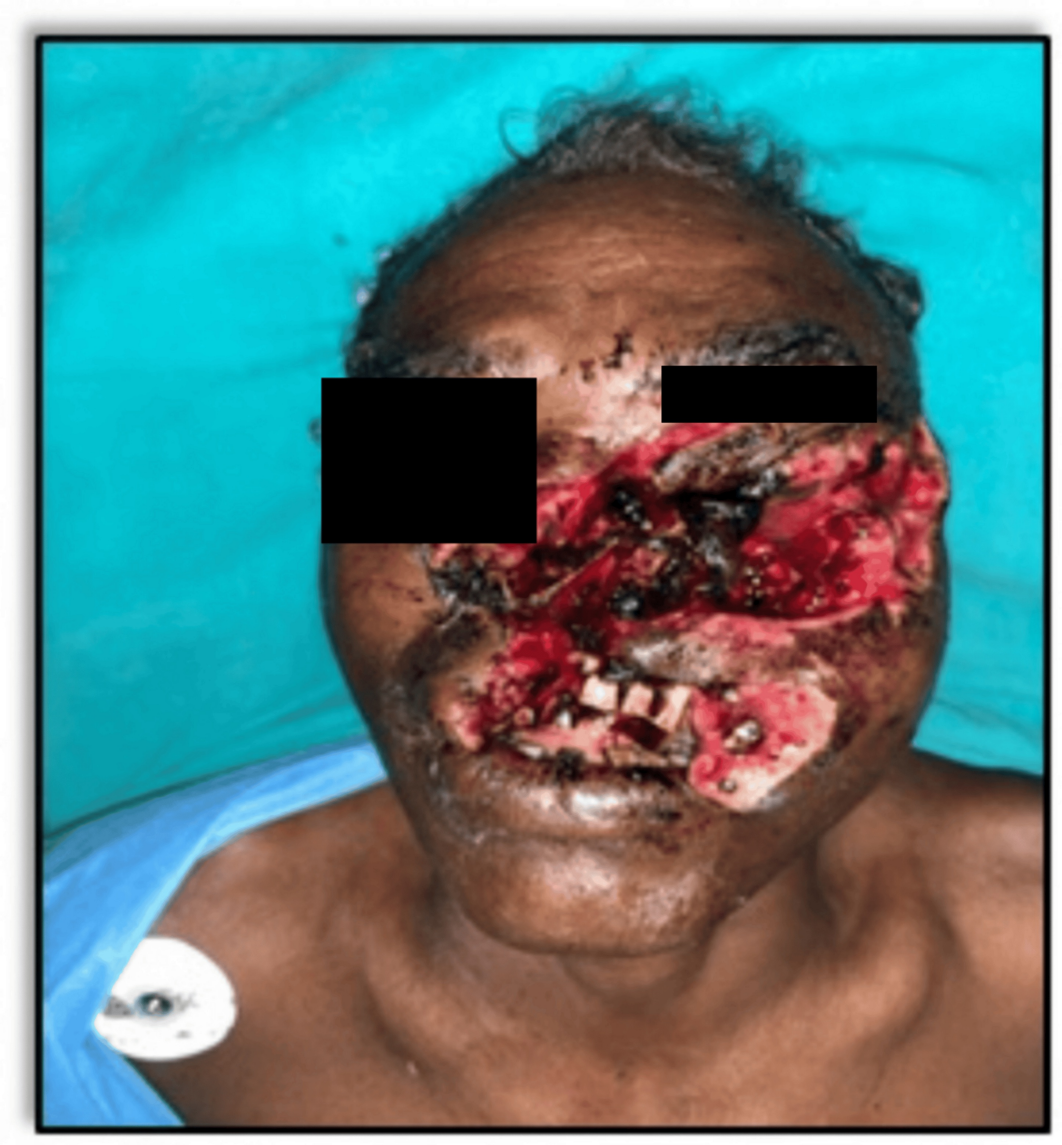
Extensive facial injuries (Case 3)

A 56-year-old male patient, with a history of hypothyroidism, presented to the trauma center following a bear bite to the face (Figure 10).

**Figure 11:**
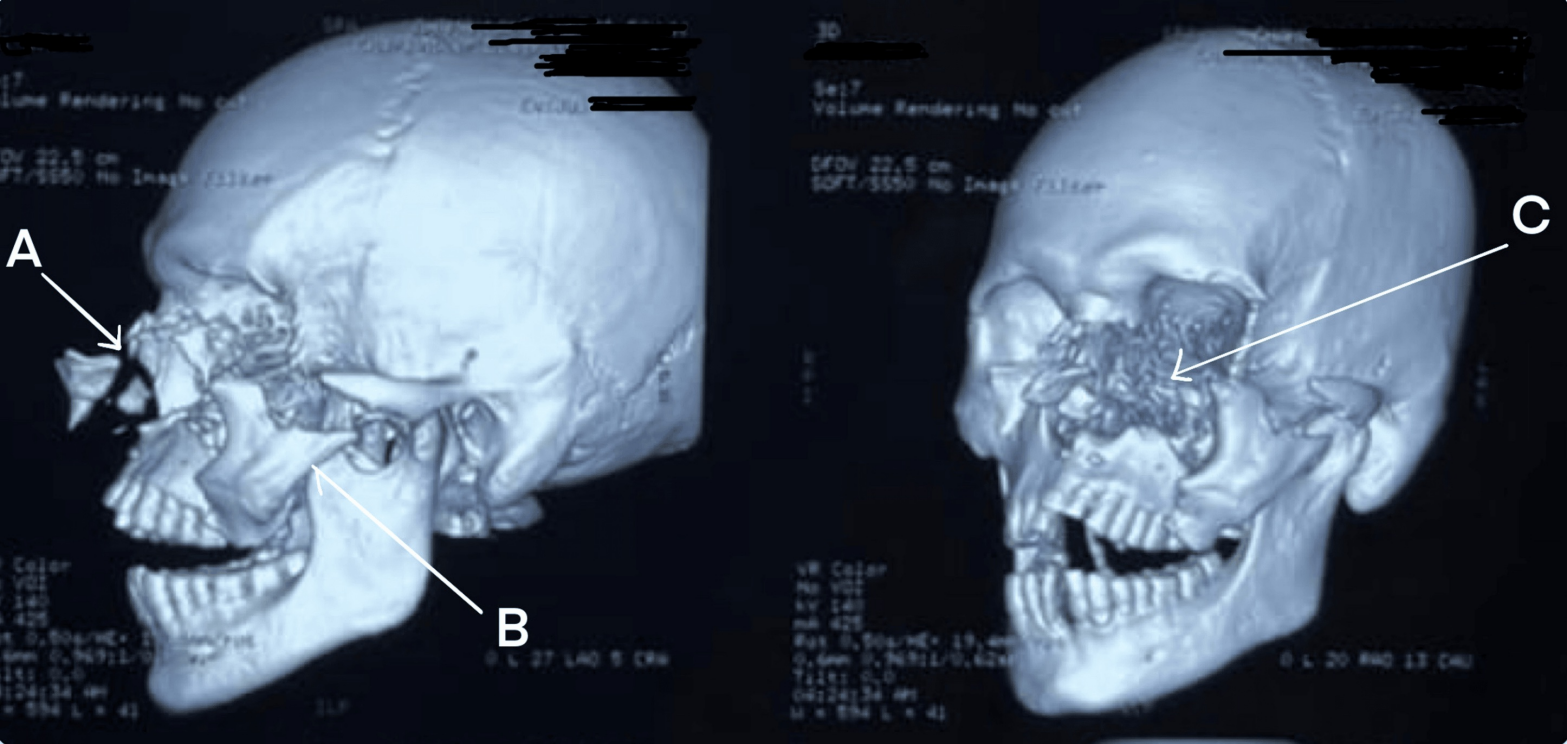
3D CT of the face and skull (Case 3) (A) Fracture of the left nasal bone. (B) Fracture of the left zygomatic process. (C) Comminuted fracture of the medial wall of the left orbit.

He complained of severe facial pain, nausea, voice change, and slight breathing difficulty. 3D CT of the face revealed extensive comminuted fractures (Figure 11).

**Figure 12:**
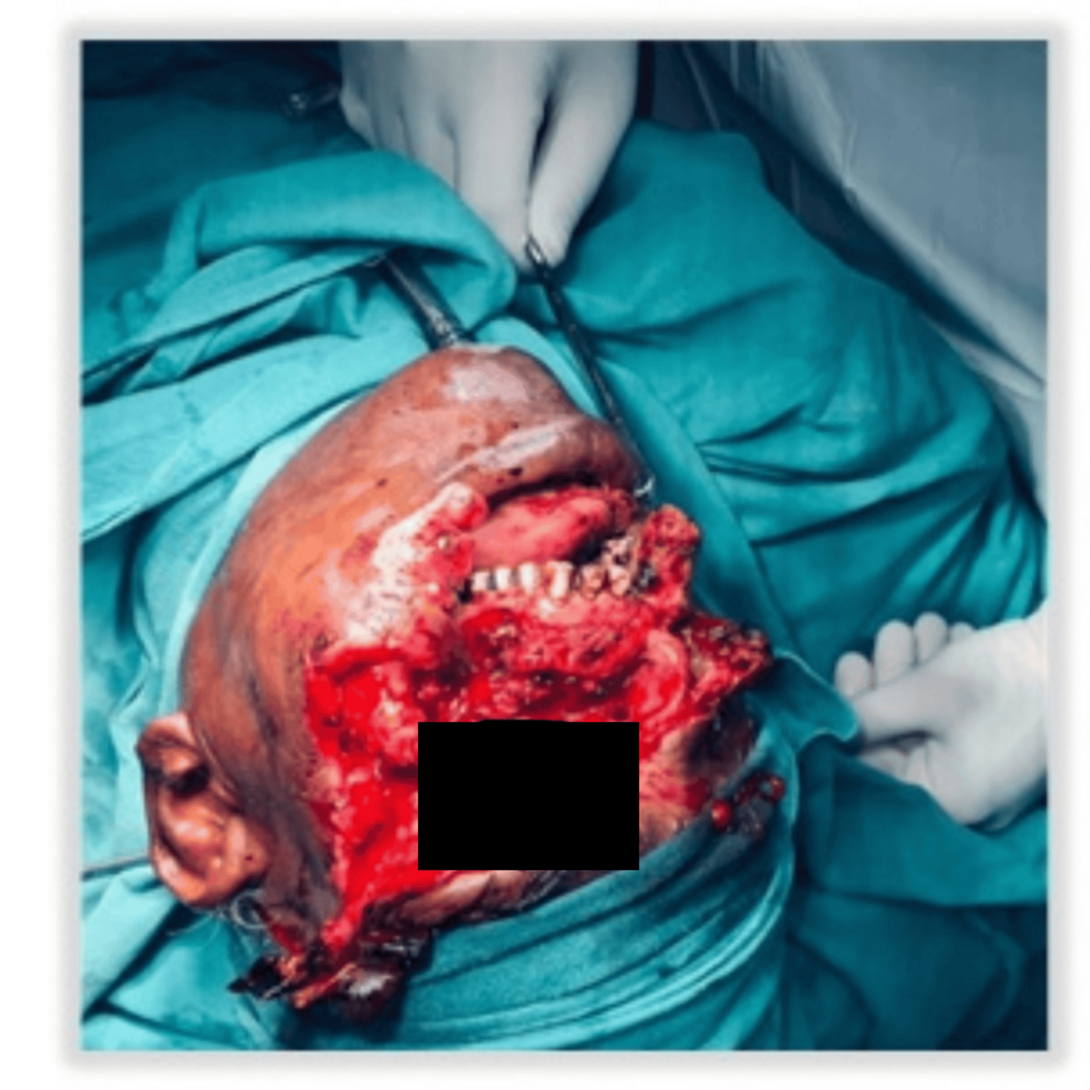
Patient with submental tube in situ (before surgery) (Case 3)

**Figure 13:**
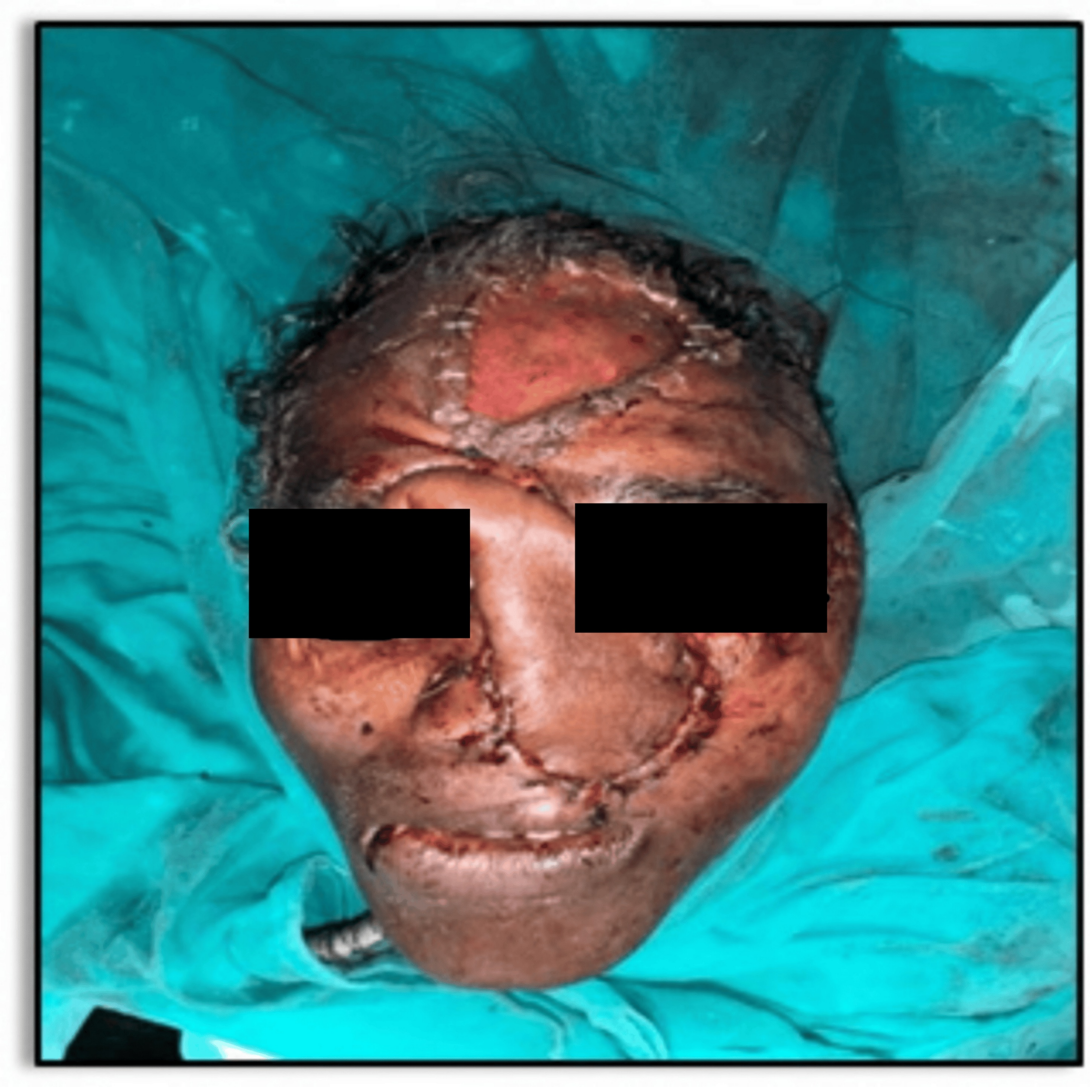
Patient with submental tube in situ (end of surgery) (Case 3)

The CT scan revealed fractures involving a displaced left nasal bone, left zygomatic process, and comminuted medial wall of the left orbit, along with the walls of the maxillary sinus and the alveolar process of the maxilla. On examination, he presented with pallor, widespread facial lacerations, degloving injury to the left temporal region, extensive periorbital bony and soft tissue injuries, and visible orbital wall, maxillary, and nasal bone fractures. Despite a patent airway, significant challenges included distorted facial anatomy, multiple loose teeth, and blood in the oral cavity, alongside a mouth opening of two fingerbreadths and normal neck motion. The patient was scheduled for ORIF under GA, with anticipated difficult airway due to the risk of bleeding and dislodgement of loose teeth. The primary airway management strategy (Plan A) was awake intubation with suction-assisted laryngoscopy and airway decontamination (SALAD) [15] via video laryngoscopy (VL) and airway adjuncts, with emergency FONA as Plan B. An awake intubation utilizing the SALAD technique with VL was successfully performed, followed by submental tube placement (Figures 12, 13).

**Figure 14:**
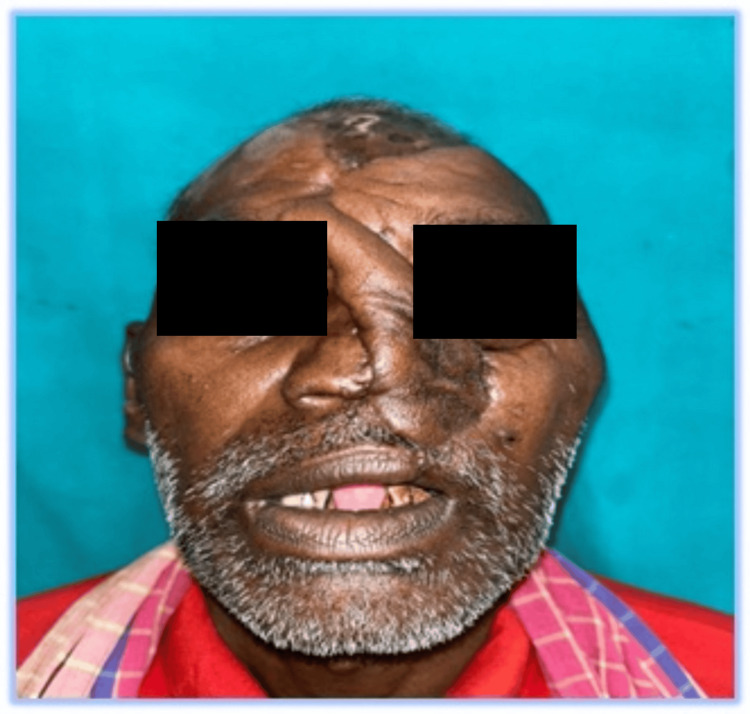
Patient at discharge (Case 3)

Prior to induction, airway point-of-care ultrasound (POCUS) was used to identify the cricothyroid membrane. Premedication and airway blocks were performed as described in Case 1. The only difference in management was the use of a suction catheter for oral decontamination and as a tongue lifter during careful VL to prevent tooth dislodgement. Further decontamination of the hypopharynx, larynx, and esophagus was performed, and the suction catheter was then repositioned into the proximal esophagus for continuous suction. The laryngoscope blade was rotated 30 degrees to the left to create a channel for ET tube passage. The airway was secured with a 7 mm cuffed flexometallic tube after optimal external laryngeal manipulation, and its position was confirmed by capnography. Propofol and vecuronium were then administered. The tube was subsequently secured submentally as described previously, with position reconfirmed by auscultation and capnography. Intraoperative anesthesia was maintained with inhalational isoflurane, 50% nitrous oxide, and 50% oxygen, along with intermittent IV vecuronium boluses. Following completion of surgery, reversal agents were administered, and the patient was transferred to the PACU for overnight mechanical ventilation due to the prolonged surgical duration. Intubation was successful, the intraoperative period was uneventful, and the patient had a good recovery and follow-up (Figure 14).

Case 4

**Figure 15:**
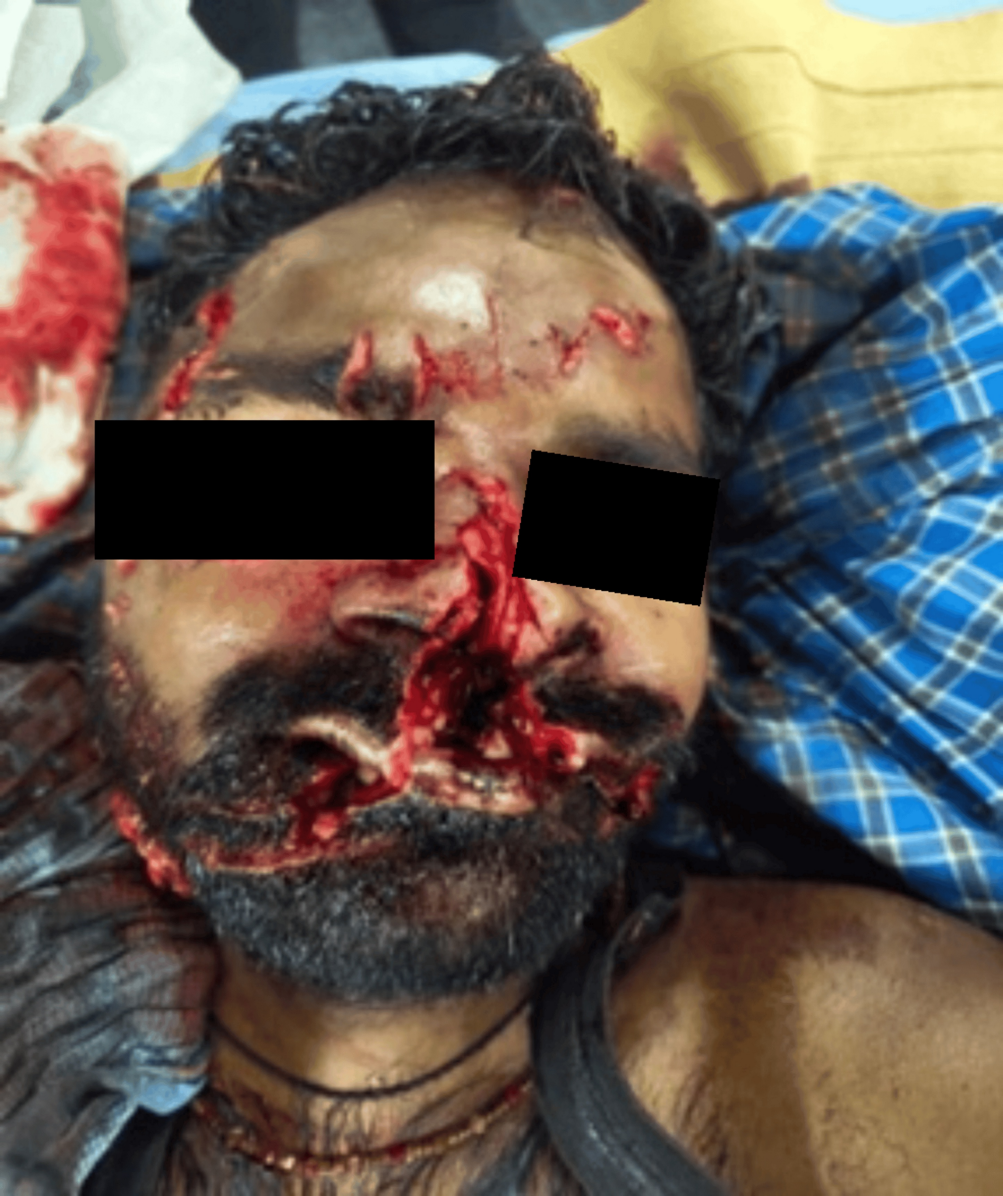
Extensive facial injuries (Case 4)

A 28-year-old male patient presented to the emergency room with an alleged history of being mauled by a bear. He had multiple facial lacerations, nasal bleeding, severe facial pain, and reduced vision in the right eye (Figure 15).

**Figure 16:**
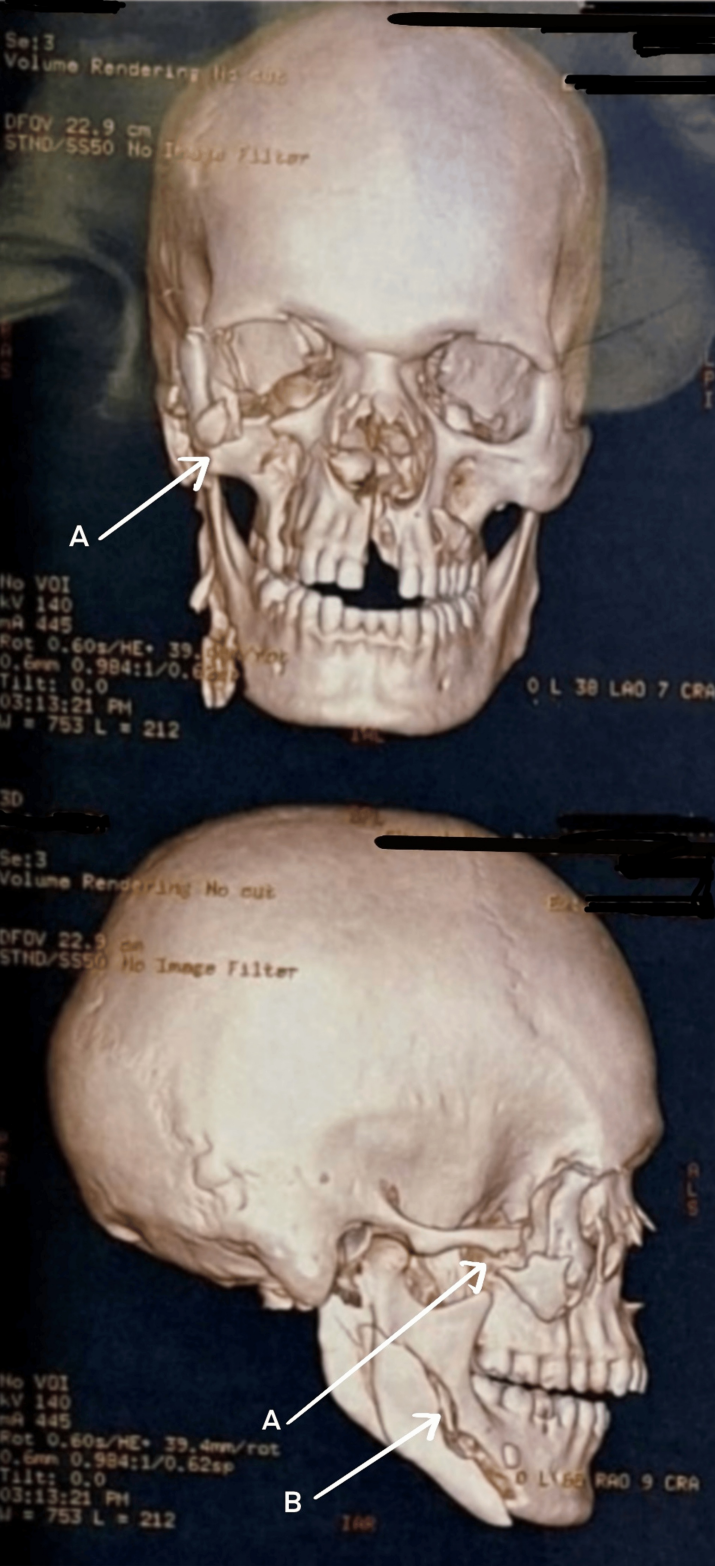
3D CT of the face and skull (Case 4) (A) Fracture of the right zygomatic complex with palatal split. (B) Fracture of the right ramus of the mandible.

**Figure 17:**
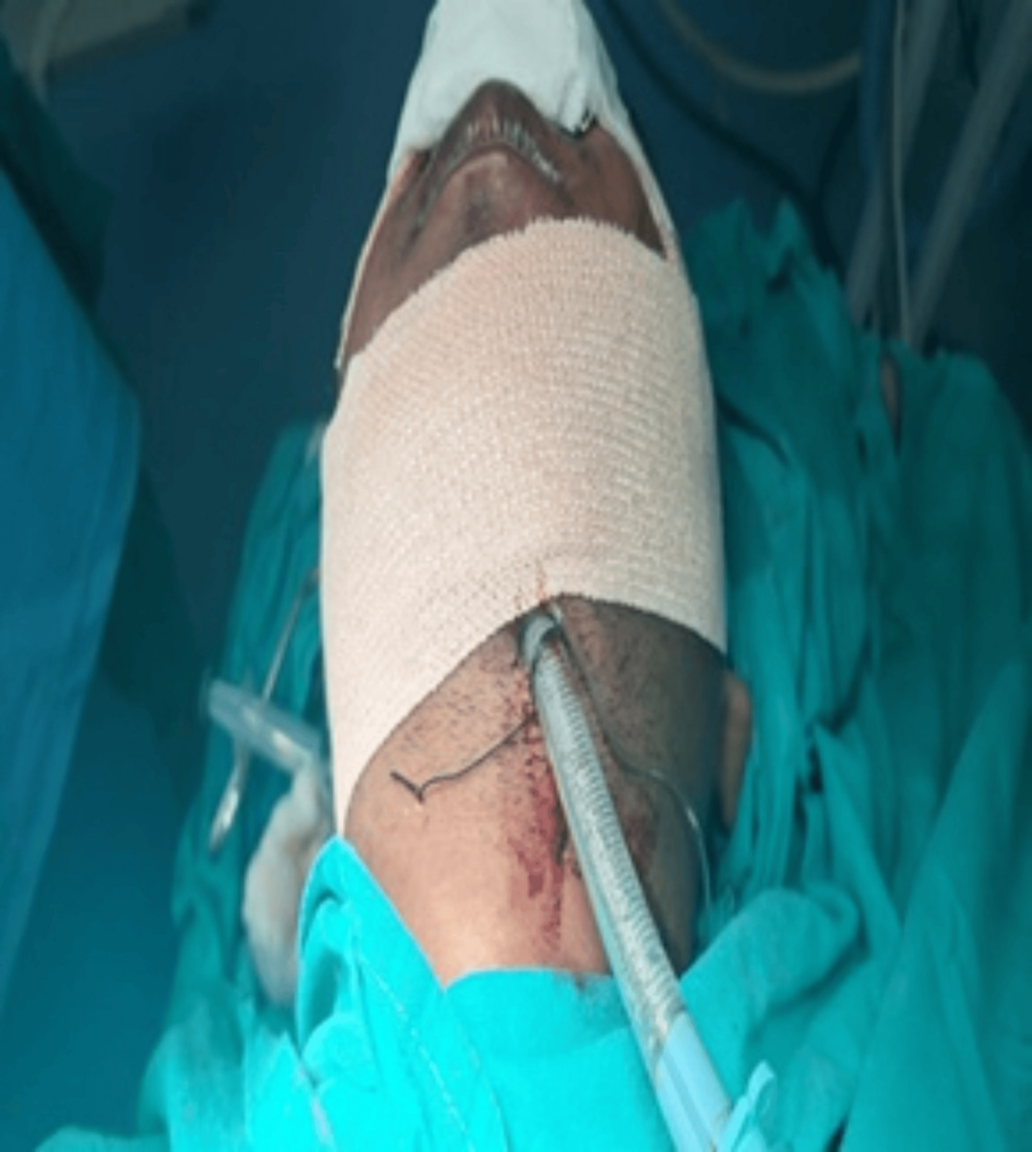
Patient with submental tube in situ (Case 4)

NCCT with 3D reconstruction revealed fracture (Figure 16) of the right ZMC with palatal split and right ramus of mandible. Systemic examination was normal, and the patient had a full GCS with a patent airway. Further examination of the airway showed a restricted mouth opening of two finger breadths, Mallampati Grade IV, multiple loose tooth, few missing teeth, and lacerations over the lower border of the mandible, medial aspect of the nose involving the upper lip and left commissure of the mouth. He was planned for ORIF under GA. The primary airway management strategy included AOFOI (Plan A) along with SALAD via VL and airway adjuncts, and emergency FONA as Plan B. Here, AOFOI was done in view of the mandibular fractures, which made the intubation via laryngoscopy difficult, so VL was used only for clearing the airway. Prior to intubation, airway POCUS was used to identify the cricothyroid membrane in case Plan B was required. This was followed by submental tube placement using technique as described earlier (Figure 17).

**Figure 18:**
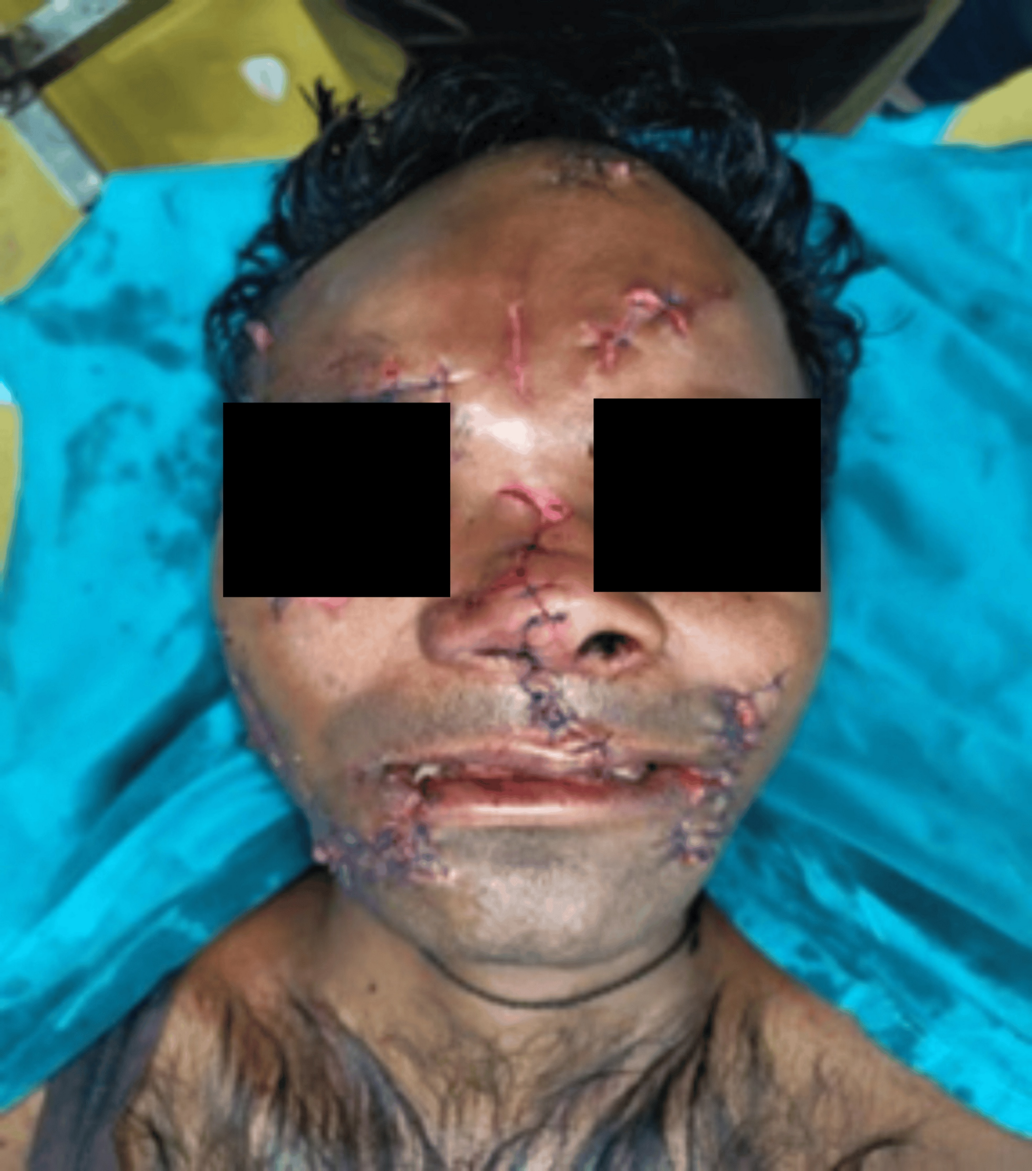
Patient post-extubation status (Case 4)

The plan for premedication, airway preparation, and subsequent anesthesia management followed the same method as in Case 1. Upon surgical completion, reversal was administered, the tube was exchanged for a PVC tube, and the patient was transferred to the PACU. The patient was extubated the following day due to the long duration of surgery and the significant risk of airway edema (Figure 18).

Case 5

**Figure 19:**
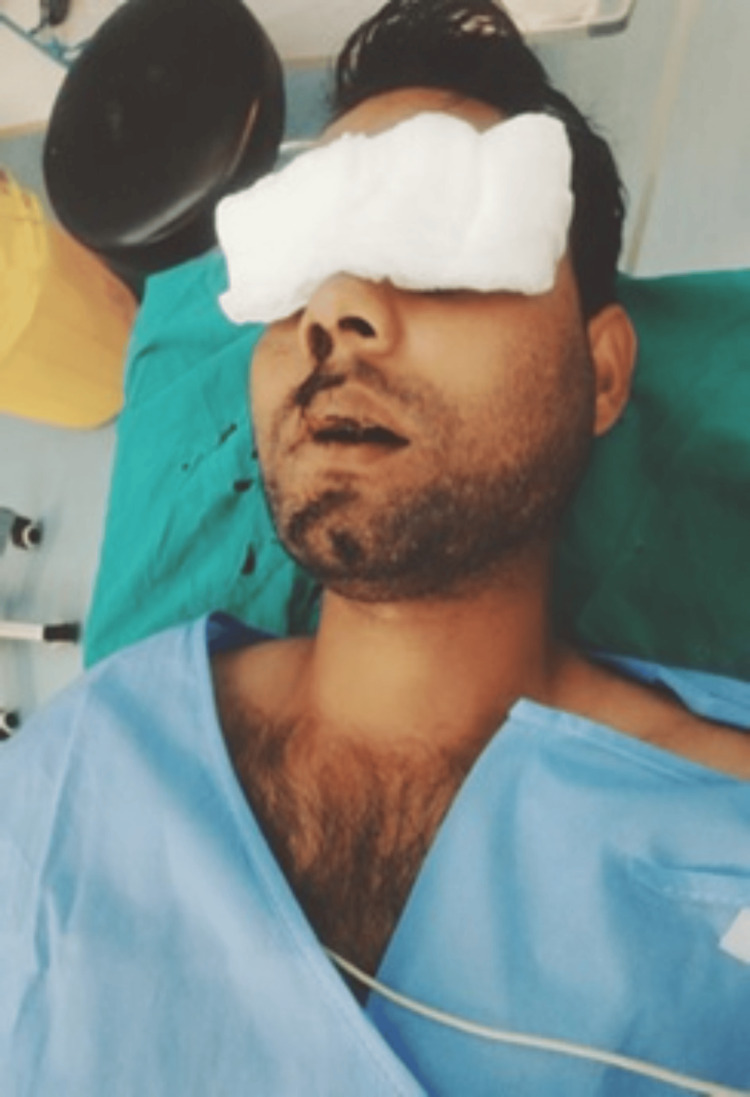
Patient status before intubation (Case 5)

A 35-year-old male patient presented to the trauma emergency with facial pain and swelling after a roadside accident (Figure 19).

**Figure 20:**
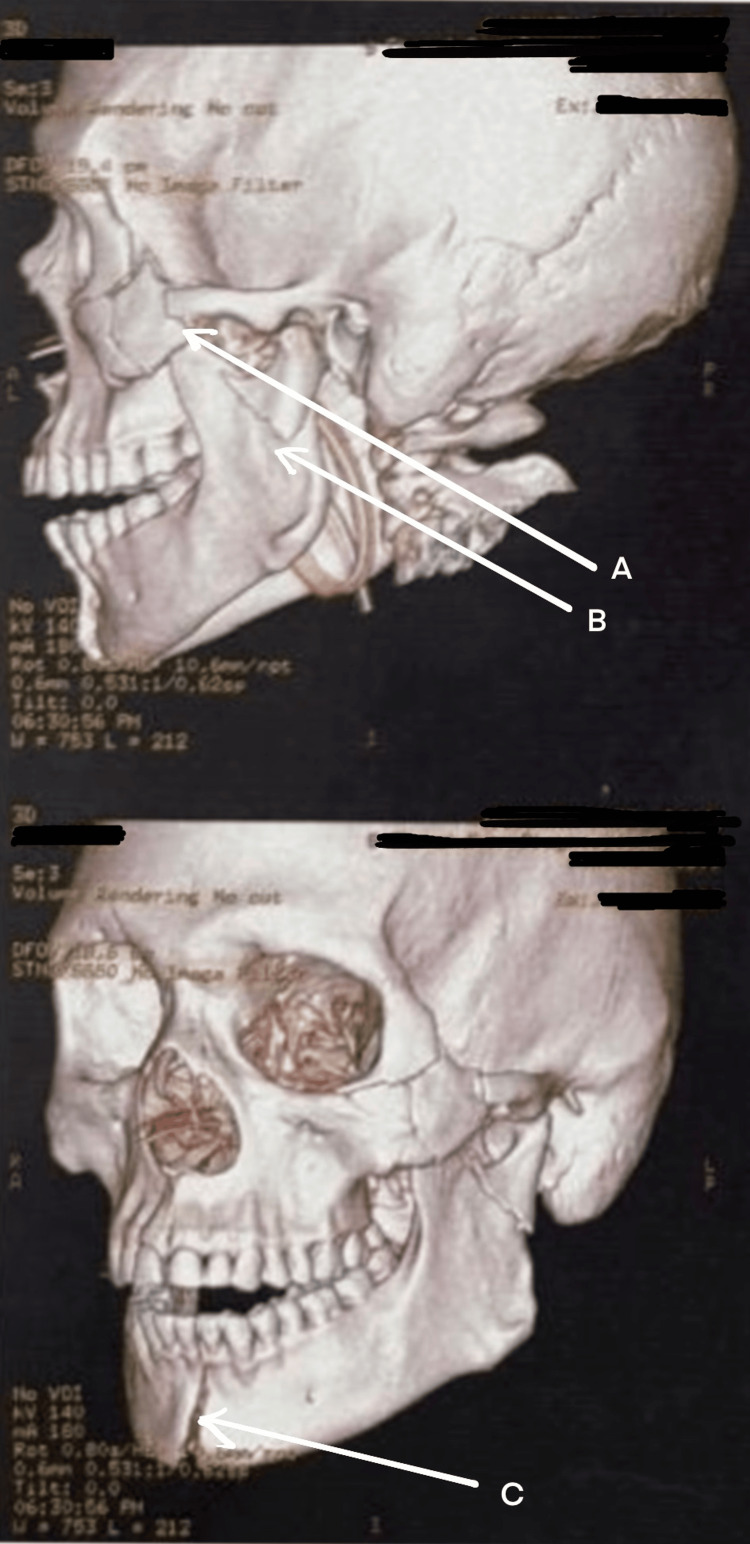
3D CT of the face and skull (Case 5) (A) Fracture of the left zygomaticomaxillary complex. (B) Fracture of the left condyle. (C) Fracture of the right mandibular parasymphysis.

After a 3D CT of the face and air sinuses was done, he was diagnosed with fractures of the left ZMC, left condyle, and the right mandibular parasymphysis (Figure 20).

On systematic examination, no significant issues were found in any major organ systems, and the GCS was E4V5M6. The airway examination revealed overall patency; however, significant facial and periorbital edema, upper lip and chin lacerations, multiple loose incisors, restricted mouth opening (1.5 finger breadths), and a Mallampati Grade IV view were noted. Neck movements were normal. He too was planned for MMF with ORIF under GA, with an anticipated difficult airway due to the risk of bleeding and loose incisor dislodgement. The airway management plan for this patient was modified to awake nasal fiberoptic-guided intubation (Plan A), and emergency FONA as Plan B. An awake nasal fiberoptic intubation was successfully performed. After proper patient counselling, oxymetazoline nasal drops were instilled in each nostril. The rest of the airway preparation and anesthesia management followed the same method as in Case 1, but here, in view of the multiple intraoral and dental injuries and to have a safer airway alternative with ease of access to the surgical field, awake nasal intubation using a 7 mm I.D. flexometallic ET tube was placed and confirmed visually and by capnography, followed by propofol and vecuronium administration. Anesthesia was maintained with isoflurane, 50% nitrous oxide, 50% oxygen, and intermittent vecuronium boluses. Upon surgical completion, reversal was administered, and the patient was extubated and transferred to the PACU. The intubation was successful, the intraoperative period was uneventful, and the patient recovered with minimal airway morbidity.

## Discussion

Table 1 summarizes the airway management in panfacial trauma of the five cases.

**Table 1:** Summary of the airway management in panfacial trauma of the five cases presented in this series AOFOI: awake oral fiberoptic intubation, ANFOI: awake nasal fiberoptic intubation, VL: video laryngoscopy, SALAD: suction-assisted laryngoscopy and airway decontamination, FZ: frontozygomatic, ZMC: zygomaticomaxillary complex, MV: mechanical ventilation, PACU: post-anesthesia care unit, POD: postoperative day, M: male. Note: All patients underwent maxillomandibular fixation (MMF) with open reduction and internal fixation (ORIF) under general anesthesia (GA). Plan B for all cases was emergency front-of-neck access (FONA). Submental intubation was performed in Cases 1-4, while nasal intubation was chosen for Case 5 due to intraoral injuries.

Case	Age/sex	Mechanism of injury	Fracture patterns	Airway challenges	Technique used	Intra- and postoperative course	Complications	Outcome
1	32/M	Road traffic accident	Bilateral LeFort II fractures, nasal process of the frontal bone, right mandibular parasymphysis, left mandibular subcondyle	Facial edema, sutured lacerations (chin & right eye), loose & missing incisors, mouth opening 1 finger breadth	AOFOI with Berman airway → submental intubation	Long-duration (~6 hrs) MMF with ORIF; intraoperative course uneventful; elective overnight MV in PACU	No airway-related or any other major complications reported	Extubated the next day; uneventful recovery and discharged in good condition
2	29/M	Road traffic accident	Right frontal bone, right FZ suture, right ZMC, right mandibular parasymphysis, left condyle	Facial & periorbital edema, lip & chin lacerations, missing incisors, mouth opening 2 finger breadths, Mallampati IV, bradycardia (HR 50-55)	AOFOI → submental intubation (no dexmedetomidine due to bradycardia)	Prolonged surgery MMF with ORIF (~7 hrs); intraoperative period uneventful; elective overnight airway protection	No significant complications; minimal airway morbidity	Extubated the following day; good postoperative recovery and discharged
3	56/M	Bear bite	Displaced left nasal bone, left zygomatic process, comminuted medial wall of the left orbit, maxillary sinus walls, alveolar process of the maxilla	Pallor, extensive facial lacerations, degloving injury (left temporal), distorted anatomy, loose teeth, blood in the oral cavity, mouth opening 2 finger breadths, hypothyroidism	Awake VL with SALAD technique → submental intubation	Prolonged ORIF (~9 hrs); anesthesia maintained with inhalational agents and muscle relaxant; elective overnight MV in PACU due to long surgery duration	No aspiration or airway failure; no major complications reported	Good recovery with satisfactory follow-up and functional outcome
4	28/M	Bear mauling	Right zygomatic complex with palatal split, right ramus of the mandible	Multiple facial lacerations, nasal bleeding, reduced vision (right eye), mouth opening 2 finger breadths, Mallampati IV, loose & missing teeth	AOFOI with SALAD via VL → submental intubation	ORIF under GA (~8 hrs); anesthesia and airway management similar to Case 1; elective overnight ventilation with delayed extubation due to edema risk and long surgery	No major airway-related or surgical complications documented	Uneventful postoperative course; extubated the next day and discharged in stable condition
5	35/M	Road traffic accident	Left ZMC, left condyle, right mandibular parasymphysis	Facial & periorbital edema, lip & chin lacerations, loose incisors, mouth opening 1.5 finger breadths, Mallampati IV	Awake nasal fiberoptic intubation (ANFOI)	MMF with ORIF under GA (~4 hrs); intraoperative course uneventful; extubated at end of surgery and transferred to PACU	No complications reported; minimal airway morbidity	Uneventful recovery and discharged with favorable outcome

Airway management in panfacial trauma is dictated by multiple factors, including injury pattern, urgency, and patient cooperation, alongside available resources and expertise. While orotracheal intubation remains the default in emergencies, nasotracheal and fiberoptic techniques are often safer when performed electively and in a planned manner. Submental intubation offers a practical and less invasive alternative to tracheostomy in surgeries requiring wide surgical access.

Cases 1 and 2 demonstrate the practical utility of submental intubation, a technique that provides unobstructed surgical access while avoiding the complications of a tracheostomy. Submental intubation has been advocated in cases where both nasal and oral placements of the tube are not feasible, like in the case of MMF [4,16].

Cases 3 and 4 demonstrate the SALAD technique, which has emerged as a valuable method for managing a contaminated airway during intubation. Its use can increase the first-pass success rates in difficult intubation and can reduce the risk of complications such as aspiration [15,17]. Used in conjunction with AOFOI, this technique helps manage extremely complicated airway scenarios, with relative safety and ease, by clearing up the vision for tube insertion. Emergency tracheostomy remains critical when standard methods fail, particularly in the presence of active bleeding, distorted anatomy, or failed visualization. Interdisciplinary planning, availability of advanced airway tools like VLs and fiberoptic bronchoscopes, and backup surgical teams are essential components of successful airway management.

Case 5 highlights the effectiveness of awake fiberoptic nasotracheal intubation. The technique is well-suited when the nasal access is feasible, allowing for a controlled, cooperative approach under topical anesthesia. Adequate preparation, including the use of topical vasopressors and sedation, can help mitigate bleeding risk and patient anxiety [18,19].

## Conclusions

Securing the airway in panfacial trauma requires a flexible, patient-specific approach. While traditional intubation techniques are often successful, submental intubation and nasotracheal intubation may provide vital alternatives to surgical tracheostomy in complex cases. This series reinforces the importance of early airway assessment, preparedness for difficult scenarios, and collaboration between surgical and anesthesia teams to ensure optimal outcomes.
